# Boundary values of Hankel and Toeplitz determinants for q-convex functions

**DOI:** 10.1016/j.mex.2024.102842

**Published:** 2024-07-03

**Authors:** Sarem H. Hadi, Timilehin Gideon Shaba, Zainab S. Madhi, Maslina Darus, Alina Alb Lupaş, Fairouz Tchier

**Affiliations:** a Department of Mathematics, College of Education for Pure Sciences, University of Basrah, Basrah 61001, Iraq; b Department of Business Management, Al-imam University College, Balad 34011, Iraq; c Department of Mathematics, Landmark University, P.M.B., Omu-Aran 1001, Nigeria; d Department of Mathematics, College of sciences, University of Basrah, Basrah 61001, Iraq; e Department of Mathematical Sciences, Faculty of Science and Technology, Universiti Kebangsaan Bangi, Selangor Darul Ehsan 43600, Malaysia; f Department of Mathematics and Computer Science, University of Oradea, 1 Universitatii Street, Oradea 410087, Romania; g Mathematics Department, College of Science, King Saud University, P.O. Box 22452, Riyadh 11495, Saudi Arabia

**Keywords:** Analytic functions, Univalent functions,, Q-calculus, Hankel determinants, Toeplitz determinants, univalent functions, q-calculus, Hankel and Toeplitz determinants, Hankel and Toeplitz determinants

## Abstract

The study of holomorphic functions has been recently extended through the application of diverse techniques, among which quantum calculus stands out due to its wide-ranging applications across various scientific disciplines. In this context, we introduce a novel q-differential operator defined via the generalized binomial series, which leads to the derivation of new classes of quantum-convex (q-convex) functions. Several specific instances within these classes were explored in detail. Consequently, the boundary values of the Hankel determinants associated with these functions were analyzed. All graphical representations and computational analyses were performed using Mathematica 12.0.

• These classes are defined by utilizing a new q-differential operator.
• The coefficient values $|a_{i}|\;\left(i=2,3,4\right)$ are investigated.
• Toeplitz determinants, such as the second $T_{2}\left(2\right)$ and the third $T_{3}\left(1\right)$ order inequalities, are calculated.

## Graphical abstract

**Figure:**
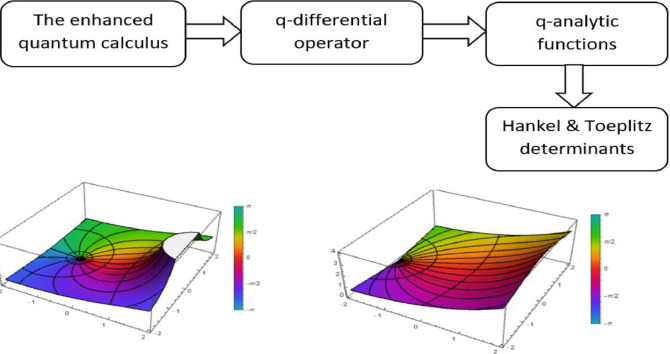

Specifications table

**Table:** 

Subject area:	*Mathematics*
More specific subject area:	*Analytic functions with*q*-calculus*
Name of your method:	*Hankel and Toeplitz determinants*
Name and reference of original method:	*The name of the method is: Hankel and Toeplitz determinants Reference* 1. *J. W. Noonan and D. K. Thomas, On the second Hankel determinant of a really mean p-valent functions, Trans. Amer. Math. Soc., 223 (2) (1976), 337–346.*https://www.ams.org/journals/tran/1976-223-00/S0002-9947-1976-0,422,607-9/ 2. M. K. Aouf, A. O. Mostafa, R. E. Elmorsy, Certain subclasses of analytic functions with varying arguments associated with q-difference operator, Afr. Mat. 32 (2021), 621–630. Link: https://link.springer.com/article/10.1007/s13370-020-00,849-3
Resource availability:	*N/A*

**Method details**

## Concepts

Let A denote the set comprising all complex-valued functions f(z) in the form

(1) $$f\left(z\right)=z+\sum_{i=2}^{\infty }\;a_{i}z^{i}$$

where these functions are analytic within the open unit disc U={z∈C:|z|<1}. A function f(z) is considered univalent within the domain U. The subset of functions in the class A that are univalent is represented as S.

A function f(z) from class A is denoted as a starlike function ($f\left(z\right)\in S^{*}$) and a convex function (f(z)∈C) if the following inequality hold:

$$ℜ\left[\frac{zf^{\prime }\left(z\right)}{f\left(z\right)}\right]>0\;\text{and}\;ℜ\left[1+\frac{zf^{\prime \prime }\left(z\right)}{f^{\prime }\left(z\right)}\right]>0,$$

where z∈U.

The sets $S^{*}\left(\lambda \right)$ and C(λ), which represent starlike and convex functions of order λ (where 0≤λ<1), are provided as follows:

$$S^{*}\left(\lambda \right)=\left\{f\left(z\right)\in A:ℜ\left\{\frac{zf^{\prime }\left(z\right)}{f\left(z\right)}\right\}>\lambda ,\;\left(z\in U\right)\right\}$$

and

$$C\left(\lambda \right)=\left\{f\left(z\right)\in A:ℜ\left\{1+\frac{zf^{\prime \prime }\left(z\right)}{f^{\prime }\left(z\right)}\right\}>\lambda ,\;\left(z\in U\right)\right\}.$$

Setting λ=0, it is evident that $S^{*}\left(0\right)=S^{*}$ and C(0)=C.

Kanas and Wisniowska [[1], [2]] defined the categories $r-U_{CV}$ and $r-U_{S^{*}}$ of r-uniformly convex functions and r-uniformly starlike functions, respectively, in the following ways:

$$r-U_{CV}=\left\{f\in A:r\left|\frac{{\left(zf^{\prime }\left(z\right)\right)}^{\prime }}{f^{\prime }\left(z\right)}-1\right|<ℜ\left(\frac{{\left(zf^{\prime }\left(z\right)\right)}^{\prime }}{f^{\prime }\left(z\right)}\right),\;z\in U\;r\geq 0\right\}$$

and

$$r-U_{S^{*}}=\left\{f\in A:r\left|\frac{zf^{\prime }\left(z\right)}{f\left(z\right)}-1\right|<ℜ\left(\frac{zf^{\prime }\left(z\right)}{f\left(z\right)}\right),\;z\in U\;r\geq 0\right\}.$$

These two categories represent extensions of the sets of convex univalent functions and uniformly starlike functions, as provided by Goodman [3]. Similarly, Liu et al. [4] explored the subfamilies $S^{*}\left(\eta ,\lambda \right)$ and C(η,λ) of analytic functions, formulated by the following inequalities:

$$\left|\frac{zf^{\prime }\left(z\right)}{f\left(z\right)}-1\right|<\lambda \left|\eta \frac{zf^{\prime }\left(z\right)}{f\left(z\right)}+1\right|,\;z\in U$$

and

$$\left|\frac{{\left(zf^{\prime }\left(z\right)\right)}^{\prime }}{f^{\prime }\left(z\right)}-1\right|<\lambda \left|\eta \frac{{\left(zf^{\prime }\left(z\right)\right)}^{\prime }}{f^{\prime }\left(z\right)}+1\right|,\;z\in U,$$

where 0<η≤1 and 0<λ≤1.

## Background

**Fig. 1:**
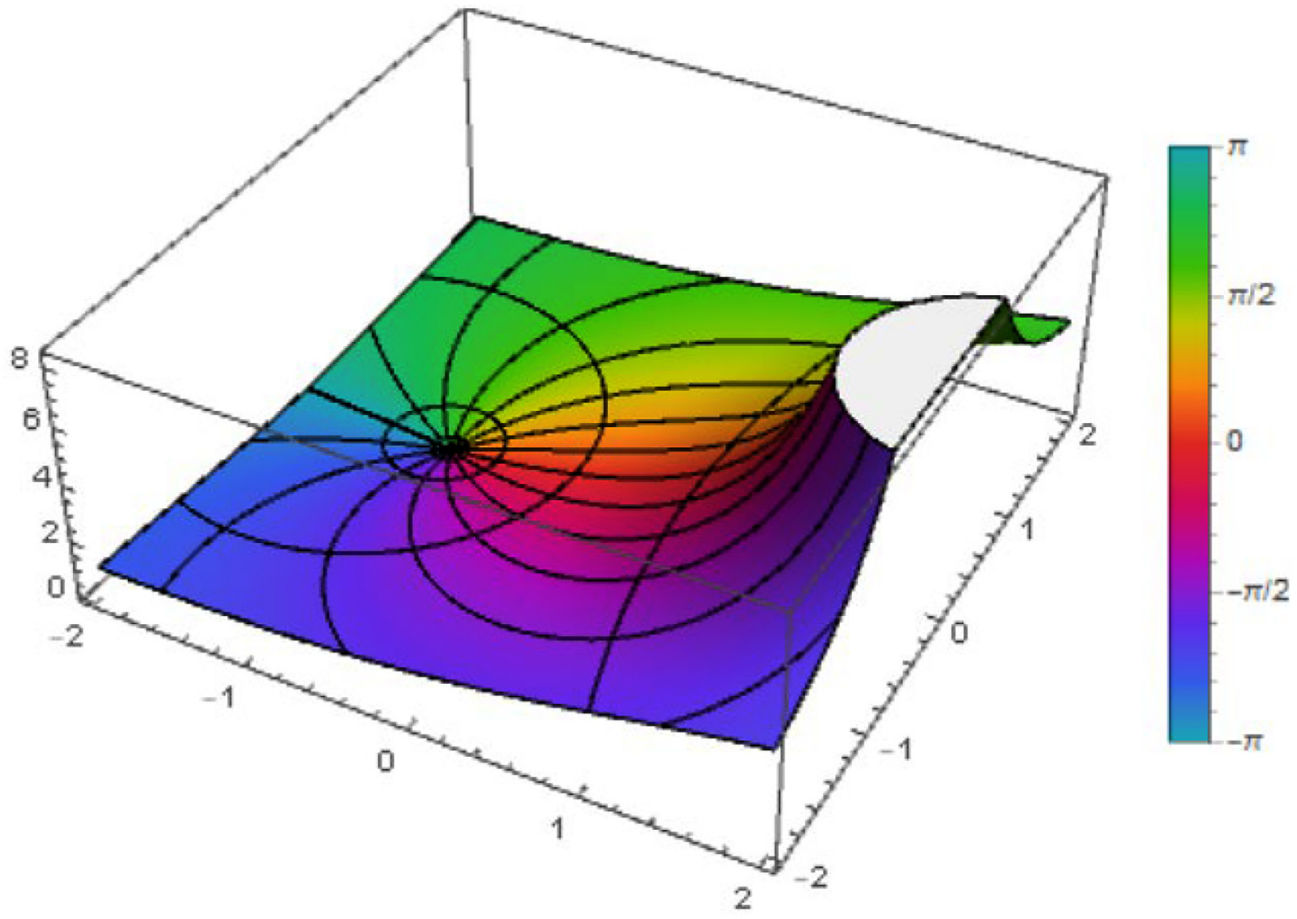
The figure shows the function ${ℵ}_{1}\left(z\right)$ with $q=\frac{1}{2}.$

**Fig. 2:**
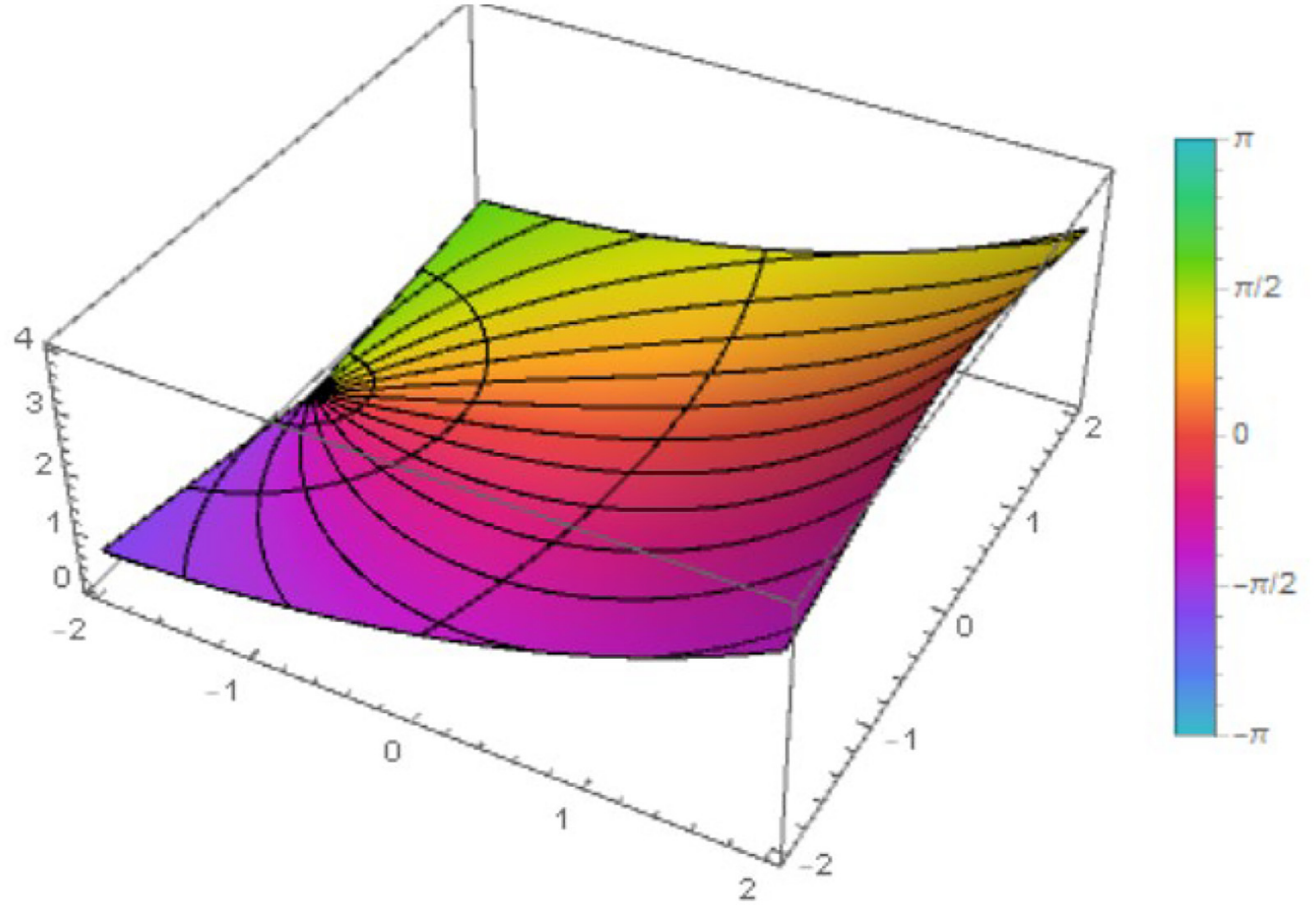
The figure shows the function ${ℵ}_{2}\left(z\right)$ with $\lambda =\eta =\frac{1}{2}.$

**Fig. 3:**
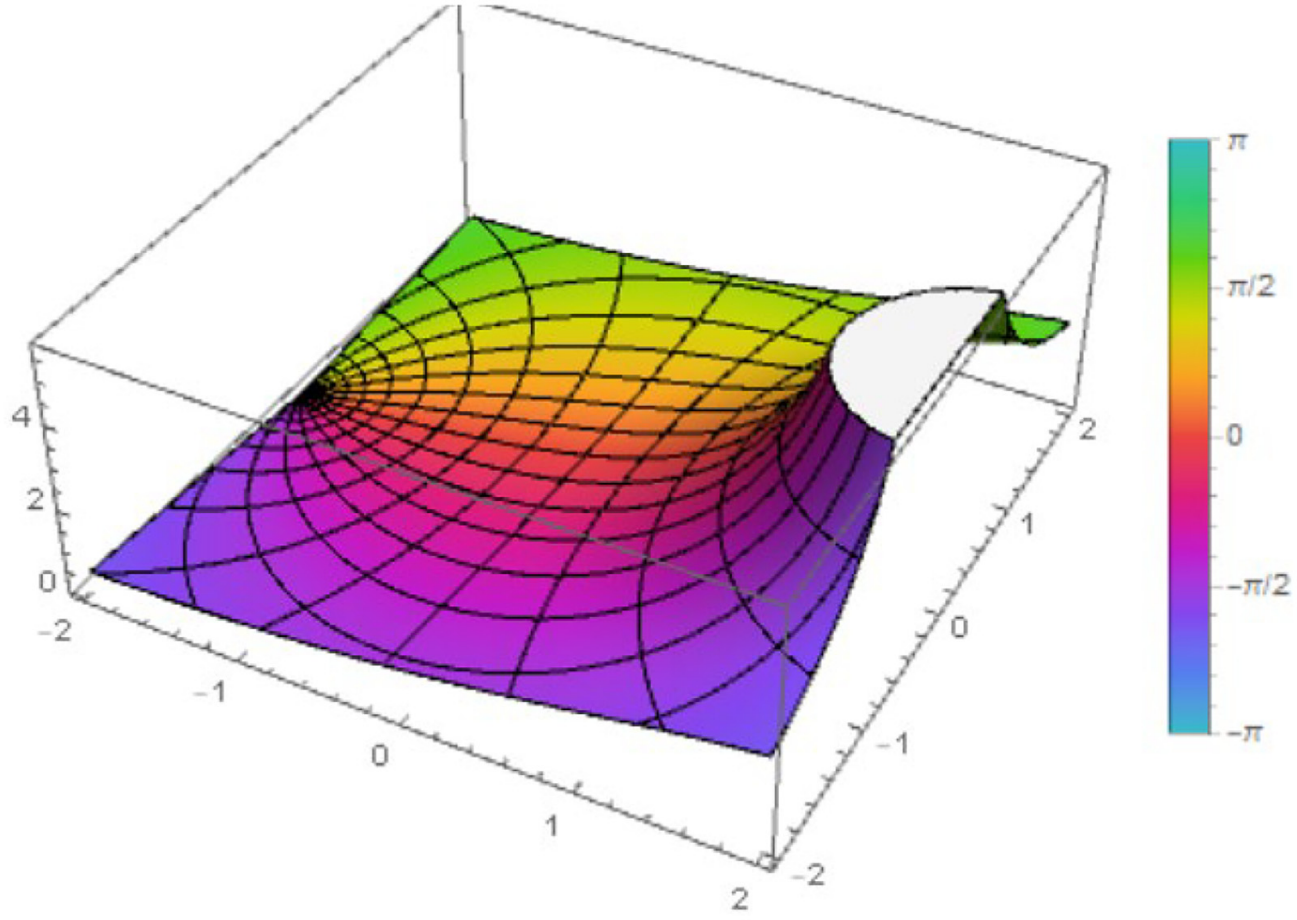
The figure shows the function ${ℵ}_{2}\left(z\right)$ with λ=1 and $\eta =\frac{1}{2}.$

**Fig. 4:**
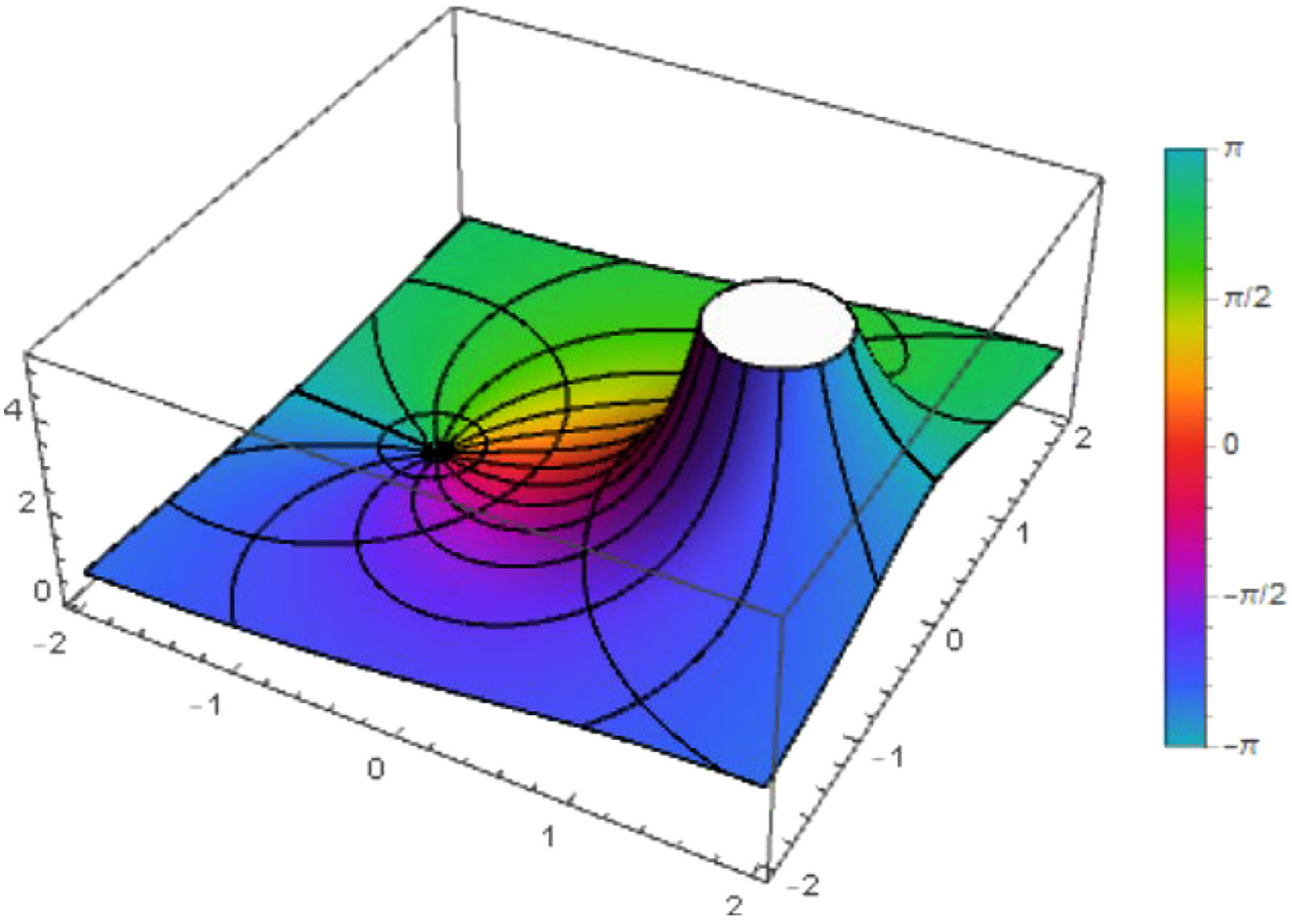
The figure shows the function ${ℵ}_{2}\left(z\right)$ with λ=η=1.

Analysis of integral and differential operators has been a fruitful field of research sice the beginning of the theory of analytic functions. The first integral operator was introduced in 1915 and is credited to Alexander [5]. Numerous viewpoints have been examined concerning these operators, including how they integrate with quantum mathematics. The study of q-calculus has become more important recently because of its extensive use in the practical sciences. Jackson [[6], [7]] pioneered the use of q-calculus to define q-analogues of derivatives. Ismail et al. [8] developed and studied q-starlike functions by using q-derivatives, which encourages more research on q-calculus in the field of geometric functions theory (GFT). As a result, many expansions of integral and differential operators using the variable q have been developed. Kanas and Raducanu [9] proposed the q-Ruscheweyh differential operator, while Noor et al. [10] investigated the q-Bernardi integral operator. In addition, Govindaraj and Sivasubramanian [11] introduced the q-Sălăgean operator as a q-analogue of the operator described in [12]. Contributions from authors [[13], [14], [15], [16], [17]] have made major advancements in the q-generalizations of certain categories of analytic functions. In a recent publication, Srivastava [18] presented a thorough review article that serves as a significant reference for researchers and academics working in the fields of generalized fractional calculus and q-calculus (Fig. 1, Fig. 2, Fig. 3, Fig. 4).

*Let us now consider certain definitions and nuances of calculus. This review will help us better understand the content presented in this new article.*

Definition 1 **[6]**

*The* q *-number* ${\left[t\right]}_{q}$ *, where* 0<q<1 *, is expressed as* $\frac{1-q^{t}}{1-q}$ *, with* t *being a complex number (* t∈C *). Specifically, when* t *is a non-negative integer, denoted as* m∈N={1,2,3,…} *,* ${\left[m\right]}_{q}$ *is defined as the sum of* q *to the power of* i *. The* n *th* q *-factorial, denoted as* ${\left[m\right]}_{q}!$ *, is defined as the product of* $\prod_{i=1}^{m}\;{\left[i\right]}_{q}$ *. For* n=0 *,* ${\left[0\right]}_{q}!$ *is set to 1.*

Definition 2. [6] *For any complex number* t*, the* q*-extended Pochhammer symbol* ${\left[t\right]}_{qm}$ *is defined as follows:*

$\text{When}\;m=0,\;{\left[t\right]}_{q0}=1.$

For natural numbers m:

$${\left[t\right]}_{qm}={\left[t\right]}_{q}{\left[t+1\right]}_{q}{\left[t+2\right]}_{q}\ldots {\left[t+m-1\right]}_{q}.$$

Definition 3. [7] *The* q*-difference operator* $D_{q}:A\to A$*, acting on* f∈A*, is described as:*

(2) $$\left(D_{q}f\right)\left(z\right)=\left\{ \begin{array}{cc} \frac{f\left(z\right)-f\left(qz\right)}{z-qz}, & \text{if}\;z\neq 0\\ f^{\prime }\left(0\right), & \text{if}\;z=0. \end{array}\right.$$

The q-derivative of f(z) appearing in (1) is as follows:

(3) $$\left(D_{q}f\right)\left(z\right)=1+\sum_{i=2}^{\infty }\;{\left[i\right]}_{q}a_{i}z^{i-1}.$$

It is worth noting that

$$\underset{q\to 1-}{\text{lim}}{\left[m\right]}_{q}=m.$$

Additionally, using a simple computation, we obtain the following operation rules for two real-valued functions, f(ξ) and g(ξ).

$$\begin{array}{ccc} & D_{q}\left(\alpha f\left(\xi \right)\pm \beta g\left(\xi \right)\right)=\alpha D_{q}f\left(\xi \right)\pm \beta D_{q}g\left(\xi \right),\alpha ,\beta \in R, & \\ & D_{q}\left(f\left(\xi \right)g\left(\xi \right)\right)=g\left(t\right)D_{q}f\left(\xi \right)+f\left(q\xi \right)D_{q}g\left(\xi \right), & \\ & D_{q}\left(\frac{f\left(\xi \right)}{g\left(\xi \right)}\right)=\frac{g\left(\xi \right)D_{q}f\left(\xi \right)-f\left(\xi \right)D_{q}g\left(\xi \right)}{g\left(\xi \right)g\left(q\xi \right)},g\left(\xi \right)\neq 0,g\left(q\xi \right)\neq 0. \end{array}$$

*Definition 4. [* 8 *] A function* f(z) *in (1.1) belongs to the class* $C_{q}$ *if the subsequent inequality holds:*

(4) $$\left|\frac{D_{q}\left(zD_{q}f\left(z\right)\right)}{D_{q}f\left(z\right)}-\frac{1}{1-q}\right|\leq \frac{1}{1-q}\;\left(z\in U\right).$$

Obviously, if q→1− then

$$\left|\frac{zf^{\prime \prime }\left(z\right)}{f^{\prime }\left(z\right)}-\frac{1}{1-q}\right|\leq \frac{1}{1-q}.$$

Alternatively, we can consider the principle of subordination functions to obtain

$$\frac{D_{q}\left(zD_{q}f\left(z\right)\right)}{D_{q}f\left(z\right)}≺{ℵ}_{1}\left(z\right),$$

where ${ℵ}_{1}\left(z\right)=\frac{1+z}{1-qz}.$

*Definition 5. For* 0<η≤1 *and* 0<λ≤1 *, we provide the subsequent uniformly* q *-convex class* $C_{q}\left(\eta ,\lambda \right)$ *of order* λ

$$\left|\frac{D_{q}\left(zD_{q}f\left(z\right)\right)}{D_{q}f\left(z\right)}-1\right|\langle \lambda |\frac{\eta D_{q}\left(zD_{q}f\left(z\right)\right)}{D_{q}f\left(z\right)}+1|.$$

Equivalently,

$$\frac{D_{q}\left(zD_{q}f\left(z\right)\right)}{D_{q}f\left(z\right)}≺{ℵ}_{2}\left(z\right),$$

where ${ℵ}_{2}\left(z\right)≔\frac{1+\lambda z}{1-\eta \lambda z},\;0<\eta \leq 1$ and 0<λ≤1.

When q→1−, it achieves the class C(η,λ) explored by Liu et al. [4].

## Motivation and research objective

Recent advancements in the study of holomorphic functions have led to significant generalizations, employing diverse techniques. Notably, the application of a specific type of quantum calculus, renowned for its wide-ranging implications across various scientific disciplines, has emerged as a prominent approach. Through these generalizations, we aim to establish novel classes of quantum-convex (q-convex) functions, characterized by the incorporation of a novel q-differential operator. Our investigation extends to the exploration of coefficient values $|a_{i}|\;\left(i=2,3,4\right)$, and we use these values in the analysis of Hankel determinants across different orders. Additionally, our study delves into specific instances of Toeplitz determinants, such as the second-order $T_{2}\left(2\right)$ and third-order $T_{3}\left(1\right)$ inequalities, providing a comprehensive exploration of these mathematical entities.

Employing the q-calculus, Shaba et al. [19] established the q-differential operator $F_{\zeta ,r\varepsilon ,\mu }^{n,\rho ,\nu ,q}:A\to A$, which is provided by

(5) $$F_{\zeta ,r\varepsilon ,\varpi }^{n,\rho ,\nu ,q}f\left(z\right)=z+\sum_{i=2}^{\infty }\;\tau_{i}a_{i}z^{i}$$

with

$$\tau_{i}≔{\left(1+\rho \left(B_{q}^{r}\left(\zeta \right)-\nu \right)\left({\left[i\right]}_{q}+\varpi -\varepsilon -1\right)\right)}^{n},\;\left(\rho >0,\nu \geq 0,\zeta >0,0\leq \varepsilon \leq \varpi \right)$$

and the q-binomial series

$$B_{q}^{r}\left(\zeta \right)=\sum_{i=1}^{r}\;{\left(\begin{array}{c} r\\ i \end{array}\right)}_{q}{\left(-1\right)}^{i+1}\zeta^{i}.$$

Remark 1**.** Several remarkable operators are also obtained (see the illustration and the references [[11], [20], [21], [22], [23], [24], [25], [26], [27], [28], [29]].

We introduce two new classes of q-convex functions, $C_{q}\left(\rho ,\nu ,\zeta ,\varpi ,\varepsilon \right)$ and $C_{q}\left(\eta ,\lambda ;\rho ,\nu ,\zeta ,\varpi ,\varepsilon \right)$, utilizing the q-differential operator $F_{\zeta ,r\varepsilon ,\mu }^{n,\rho ,\nu ,q}f\left(z\right).$

*Definition 6. For*0<q<1*, we call*f(z)*in*$C_{q}\left(\rho ,\nu ,\zeta ,\varpi ,\varepsilon \right)$*, if and only if*

$$\left|\frac{D_{q}\left(zD_{q}F_{\zeta ,r\varepsilon ,\varpi }^{n,\rho ,\nu ,q}f\left(z\right)\right)}{D_{q}F_{\zeta ,r\varepsilon ,\varpi }^{n,\rho ,\nu ,q}f\left(z\right)}-\frac{1}{1-q}\right|\leq \frac{1}{1-q}$$

or equivalently

(6) $$\frac{D_{q}\left(zD_{q}F_{\zeta ,r\varepsilon ,\varpi }^{n,\rho ,\nu ,q}f\left(z\right)\right)}{D_{q}F_{\zeta ,r\varepsilon ,\varpi }^{n,\rho ,\nu ,q}f\left(z\right)}≺{ℵ}_{1}\left(z\right),\;\left({ℵ}_{1}\left(z\right)=\frac{1+z}{1-qz}\right).$$

(ρ>0,ν≥0,ζ>0,0≤ε≤ϖ,andz∈U).

*Definition 7. For* 0<η≤1 *and* 0<λ≤1 *, we call* f(z) *in* $C_{q}\left(\eta ,\lambda ;\rho ,\nu ,\zeta ,\varpi ,\varepsilon \right)$ *, if and only if*

(7) $$\frac{D_{q}\left(zD_{q}F_{\zeta ,r\varepsilon ,\varpi }^{n,\rho ,\nu ,q}f\left(z\right)\right)}{D_{q}F_{\zeta ,r\varepsilon ,\varpi }^{n,\rho ,\nu ,q}f\left(z\right)}≺{ℵ}_{2}\left(z\right),\;\left({ℵ}_{2}\left(z\right)=\frac{1+\lambda z}{1-\eta \lambda z}\right).$$

This class is equivalent to

$$\left|\frac{D_{q}\left(zD_{q}F_{\zeta ,r\varepsilon ,\varpi }^{n,\rho ,\nu ,q}f\left(z\right)\right)}{D_{q}F_{\zeta ,r\varepsilon ,\varpi }^{n,\rho ,\nu ,q}f\left(z\right)}-1\right|\langle \lambda \left|\frac{\eta D_{q}\left(zD_{q}F_{\zeta ,r\varepsilon ,\varpi }^{n,\rho ,\nu ,q}f\left(z\right)\right)}{D_{q}F_{\zeta ,r\varepsilon ,\varpi }^{n,\rho ,\nu ,q}f\left(z\right)}+1\right|.$$

**Remark 2.2** *It is evident from (* 6 *) and (* 7 *) that*

1. If n=0 in the class (6) we get the original class $C_{q}$ deduced by Ismail et al. [8].
2. If q→1− and n=0 in the class (7) we get the original class C(η,λ) established by Liu et al. [4].

The $H_{l}\left(m\right)$ Hankel determinant of the function f, was introduced by Noonan and Thomas [29].

In this case, l≥1, m≥1, and $c_{1}=1$

(8) $$H_{l}\left(m\right)=\left|\begin{array}{cccc} c_{m} & c_{m+1} & \ldots & c_{m+l-1}\\ c_{m+1} & c_{m+2} & \ldots & c_{m+l}\\ \;\vdots & \;\vdots & \ddots & \;\vdots \\ c_{m+l-1} & c_{m+l} & \ldots & c_{m+2l-2} \end{array}\right|.$$

When l=2 and m=1, the Hankel determinant $H_{l}\left(m\right)$ reduces to the famous Fekete–Szegö functional:

$$H_{2}\left(1\right)=\left[\begin{array}{cc} 1 & c_{2}\\ c_{2} & c_{3} \end{array}\right]=\left|c_{3}-c_{2}^{2}\right|.$$

This functionality is additionally extended in a more generalized form as:

$$\left|c_{3}-\sigma c_{2}^{2}\right|$$

where σ represents either a real or complex number.

The importance of the Hankel determinant becomes apparent in singularity theory [30], and it is valuable for analyzing power series with integer coefficients (refer to [[4], [31]]). Numerous scholars have derived upper bounds for $H_{l}\left(m\right)$ across different combinations of m and n within various subclasses of analytic functions (see, for instance, [[32], [33], [34]]).

The symmetric Toeplitz determinant $T_{l}\left(m\right)$ is defined as follows:

(9) $$T_{l}\left(m\right)=\left|\begin{array}{cccc} c_{m} & c_{m+1} & \ldots & c_{m+l-1}\\ c_{m+1} & c_{m+2} & \ldots & c_{m+l-2}\\ \;\vdots & \;\vdots & \ddots & \;\vdots \\ c_{m+l-1} & c_{m+l-2} & \ldots & \;c_{m} \end{array}\right|$$

where l≥1, m≥1 and $c_{m}=1$, which was inferred by Thomas and Abdul Halim [35].

Recently, some scholars have worked on examining the limits of the Toeplitz determinant $T_{l}\left(m\right)$ for different analytic function families (see, e.g., [[36], [37], [38]]). Toeplitz of analytic functions introduced by means of the Borel distribution is explored in [39], whereas quantum calculus is included in the study of Toeplitz determinants carried out in [40].

The proof of our key results depends on the application of the following lemmas:

## Auxiliary lemmas

To demonstrate Hankel determinants bounds for the classes $C_{q}\left(\rho ,\nu ,\zeta ,\varpi ,\varepsilon \right)$ and $C_{q}\left(\eta ,\lambda ;\rho ,\nu ,\zeta ,\varpi ,\varepsilon \right)$, the subsequent Lemmas must first be introduced.

The familiar class of Carathéodory functions $\gamma \left(z\right)=1+\sum_{i=1}^{\infty }\;\gamma_{i}z^{i}$ with R(γ(z))>0 is pointed by P.

Lemma 1. *[*41*] If the function* γ(z)∈P*, then*

$$\left|\gamma_{i}\right|\leq 2,\;\left(i\geq 2\right).$$

Lemma 2. *[*26*] If the function* γ(z)∈P*, then*

$$2\gamma_{2}=\gamma_{1}^{2}+\mu \left(4-\gamma_{1}^{2}\right)$$

and

$$4\gamma_{3}=\gamma_{1}^{3}+2\left(4-\gamma_{1}^{2}\right)\gamma_{1}\mu -\left(4-\gamma_{1}^{2}\right)\gamma_{1}\mu^{2}+2\left(4-\gamma_{1}^{2}\right)\left(1-|\mu {|}^{2}\right)z$$

with |μ|≤1 and |z|≤1, for some μ and z.

Lemma 3. *[*42*] If the function* γ(z)∈P*, then*

$$\left|\gamma_{2}-\sigma \gamma_{1}^{2}\right|\leq 2\text{max}\left(1,\left|2\sigma -1\right|\right),\;\left(\sigma \in C\right).$$

Subsequently, we should beging by investigating the Hankel determinant of first type $H_{2}\left(1\right)$ in order to find the maximum value of the 2th-order Hankel determinant $H_{2}\left(2\right)$.

## Method validation

We shall identify initial coefficients bounds in the following theorem, which will aid in the proof of objective findings.

*Theorem 1. If* $f\left(z\right)\in C_{q}\left(\rho ,\nu ,\zeta ,\varpi ,\varepsilon \right)$ *, with* f(z) *as in (1.1) then*

$$H_{2}\left(1\right)=\left|a_{3}-a_{2}^{2}\right|\leq \frac{1}{2q\left(1+q+q^{2}\right)\tau_{3}}.$$

*Proof.* Considering the subordination requirement expressed in Eq. (6) we obtain

(10) $$\frac{D_{q}\left(zD_{q}F_{\zeta ,r\varepsilon ,\varpi }^{n,\rho ,\nu ,q}f\left(z\right)\right)}{D_{q}F_{\zeta ,r\varepsilon ,\varpi }^{n,\rho ,\nu ,q}f\left(z\right)}={ℵ}_{1}\left(\omega \left(z\right)\right).$$

We will now proceed to demonstrate the function γ(z) in the following method:

$$\gamma \left(z\right)=\frac{1+\omega \left(z\right)}{1-\omega \left(z\right)}=1+\gamma_{1}z+\gamma_{2}z^{2}+\gamma_{3}z^{3}+\gamma_{4}z^{4}+\ldots_{.}$$

Obviously, γ∈P, it achieves

$$\omega \left(z\right)=\frac{\gamma \left(z\right)-1}{\gamma \left(z\right)+1}$$

and

$${ℵ}_{1}\left(\omega \left(z\right)\right)=\frac{2\gamma \left(z\right)}{1+\left(1-q\right)\gamma \left(z\right)+q}.$$

A calculation produces

$$\begin{array}{ccc} \frac{2\gamma \left(z\right)}{1+\left(1-q\right)\gamma \left(z\right)+q} & = & 1+\frac{\left(1+q\right)\gamma_{1}}{2}z+\left\{\frac{\left(1+q\right)\gamma_{2}}{2}-\frac{\left(1-q^{2}\right)\gamma_{1}^{2}}{4}\right\}z^{2}\;+\left\{\frac{\left(1+q\right)\gamma_{3}}{2}-\frac{\left(1-q^{2}\right)\gamma_{1}\gamma_{2}}{2}+\frac{\left(1+q\right){\left(1-q\right)}^{2}\gamma_{1}^{3}}{8}\right\}z^{3}\\ & & +\;\{\frac{\left(1+q\right)\gamma_{4}}{2}+\frac{\left(1-q^{2}\right)\gamma_{2}^{2}}{4}-\frac{\left(1-q^{2}\right)\gamma_{1}\gamma_{3}}{2}+\frac{3\left(1+q\right){\left(q-1\right)}^{2}\gamma_{1}^{2}\gamma_{2}}{8}+\frac{\left(1+q\right){\left(1-q\right)}^{3}\gamma_{1}^{4}}{16}\}z^{4}+\cdots . \end{array}$$

We derive the following from the second side of Eq. (10):

(11) $$\begin{array}{ccc} \frac{D_{q}\left(zD_{q}F_{\zeta ,r\varepsilon ,\varpi }^{n,\rho ,\nu ,q}f\left(z\right)\right)}{D_{q}F_{\zeta ,r\varepsilon ,\varpi }^{n,\rho ,\nu ,q}f\left(z\right)} & = & 1+q\left(1+q\right)a_{2}\tau_{2}z+q\left(1+q\right)\left(\left(1+q+q^{2}\right)a_{3}\tau_{3}-\left(1+q\right)a_{2}^{2}\tau_{2}^{2}\right)z^{2}\\ & & +\;q\left(1+q\right)({\left(1+q\right)}^{2}a_{2}^{3}\tau_{2}^{3}-\left(2+q\right)\left(1+q+q^{2}\right)a_{2}a_{3}\tau_{2}\tau_{3}\\ & & +\left(1+q^{2}\right)\left(1+q+q^{2}\right)a_{4}\tau_{4})z^{3}+\cdots . \end{array}$$

Compared to that, we deduce

(12) $$a_{2}=\frac{1}{2q\tau_{2}}\gamma_{1},$$

(13) $$a_{3}=\frac{1}{2q\left(1+q+q^{2}\right)\tau_{3}}\gamma_{2}+\frac{\left(1+q^{2}\right)}{4q^{2}\left(1+q+q^{2}\right)\tau_{3}}\gamma_{1}^{2}$$

and

(14) $$\begin{array}{ccc} a_{4} & = & \frac{1}{2q\left(1+q^{2}\right)\left(1+q+q^{2}\right)\tau_{4}}\gamma_{3}-\frac{q-2q^{2}-2}{4q^{2}\left(1+q^{2}\right)\left(1+q+q^{2}\right)\tau_{4}}\gamma_{1}\gamma_{2}\\ & & +\;\frac{\left(1-q+q^{2}\right)}{8q^{3}\left(1+q+q^{2}\right)\tau_{4}}\gamma_{1}^{3}. \end{array}$$

Here, the functional $|a_{3}-a_{2}^{2}|$ can be find utilizing the equations of $a_{2}$ and $a_{3}$

$$\left|a_{3}-a_{2}^{2}\left|=\frac{1}{2q\left(1+q+q^{2}\right)\tau_{3}}\right|\gamma_{2}-\left(\frac{\left(1+q+q^{2}\right)\tau_{3}-\left(1+q^{2}\right)\tau_{2}^{2}}{2q\tau_{2}^{2}}\right)\gamma_{1}^{2}\right|.$$

Considering Lemma 2 together with $\gamma_{1}\leq 2,$ we attain

$$\left|a_{3}-a_{2}^{2}\left|=\frac{1}{2q\left(1+q+q^{2}\right)\tau_{3}}\right|\frac{\mu \left(4-\gamma_{1}^{2}\right)}{2}-\left(\frac{\left(1+q+q^{2}\right)\tau_{3}-\left(1+q+q^{2}\right)\tau_{2}^{2}}{2q\tau_{2}^{2}}\right)\gamma_{1}^{2}\right|.$$

Setting $\gamma_{1}=\gamma \;\left(\gamma \in [0,2]\right)$ and |μ|=ϑ, it achieves that

(15) $$\begin{array}{c} \left|a_{3}-a_{2}^{2}\right|\leq \frac{1}{2q\left(1+q+q^{2}\right)\tau_{3}}\left(\frac{\vartheta \left(4-\gamma^{2}\right)}{2}+\left(\frac{\left(1+q+q^{2}\right)\tau_{3}-\left(1+q+q^{2}\right)\tau_{2}^{2}}{2q\tau_{2}^{2}}\right)\gamma^{2}\right)=\chi \left(\gamma ,\vartheta \right). \end{array}$$

We reach to the subsequent outcome by partially differentiating the function χ(γ,ϑ) with regard to ϑ.

$$\frac{\partial \chi \left(\gamma ,\vartheta \right)}{\partial \vartheta }>0.$$

As a consequence, the function χ(γ,ϑ) becomes an increasing function of ϑ, when ϑ is within the interval [0,1]. Therefore, the relation is satisfied by the maximal value of χ(γ,ϑ) at ϑ=1.

$$max\left\{\chi \left(\gamma ,\vartheta \right)\right\}=\chi \left(\gamma ,1\right)=C_{1}\left(\gamma \right),$$

with

$$C_{1}\left(\gamma \right)≔\frac{1}{2q\left(1+q+q^{2}\right)\tau_{3}}\left(2+\left(\frac{\left(1+q+q^{2}\right)\tau_{3}-{\left(1+q\right)}^{2}\tau_{2}^{2}}{2q\tau_{2}^{2}}\right)\gamma^{2}\right).$$

It is evident that $C_{1}\left(\gamma \right)$ permits a maximal record at γ=0; therefore, it follows that

$$\left|a_{3}-a_{2}^{2}\right|\leq C_{1}\left(\gamma \right)=\frac{1}{2q\left(1+q+q^{2}\right)\tau_{3}}.$$

*Theorem 2. If* $f\left(z\right)\in C_{q}\left(\eta ,\lambda ;\rho ,\nu ,\zeta ,\varpi ,\varepsilon \right)$ *, with* f(z) *as in (* 1 *) then*

$$H_{2}\left(1\right)=\left|a_{3}-a_{2}^{2}\right|\leq \frac{\lambda \left(1+\eta \right)}{q\left(1+q\right)\left(1+q+q^{2}\right)\tau_{3}}.$$

*Proof.* Considering the subordination requirement expressed in Eq. (7) we attain.

(16) $$\frac{D_{q}\left(zD_{q}F_{\zeta ,r\varepsilon ,\varpi }^{n,\rho ,\nu ,q}f\left(z\right)\right)}{D_{q}F_{\zeta ,r\varepsilon ,\varpi }^{n,\rho ,\nu ,q}f\left(z\right)}={ℵ}_{2}\left(\omega \left(z\right)\right).$$

After making a simplification of ${ℵ}_{2}\left(z\right)$, we obtain

(17) $${ℵ}_{2}\left(z\right)=1+\lambda \left(1+\eta \right)z+\eta \lambda^{2}\left(1+\eta \right)z^{2}+\eta^{2}\lambda^{3}\left(1+\eta \right)z^{3}+\cdots .$$

We will now illustrate the behavior of the function γ(z) using the following method:

$$\gamma \left(z\right)=\frac{1+\omega \left(z\right)}{1-\omega \left(z\right)}=1+\gamma_{1}z+\gamma_{2}z^{2}+\gamma_{3}z^{3}+\gamma_{4}z^{4}+\ldots_{.}$$

Distinctly, γ∈P then

$$\omega \left(z\right)=\frac{\gamma \left(z\right)-1}{\gamma \left(z\right)+1}=\frac{1}{2}\gamma_{1}z+\frac{1}{2}\left(\gamma_{2}-\frac{1}{2}\gamma_{1}^{2}\right)z^{2}+\frac{1}{2}\left(\gamma_{3}-\gamma_{1}\gamma_{2}+\frac{1}{4}\gamma_{1}^{3}\right)z^{3}+\cdots .$$

Considering of ${ℵ}_{2}\left(z\right)$ and γ(z), it can be inferred that

(18) $$\begin{array}{ccc} {ℵ}_{1}\left(\omega \left(z\right)\right) & = & 1+\frac{\lambda \left(1+\eta \right)}{2}\gamma_{1}z+\left\{\frac{\lambda \left(1+\eta \right)}{2}\gamma_{2}+\frac{\lambda \left(1+\eta \right)\left(\lambda \eta -1\right)}{4}\gamma_{1}^{2}\right\}z^{2}\\ & & +\;\frac{\lambda \left(1+\eta \right)}{2}\left\{\gamma_{3}+\left(\eta \lambda -1\right)\gamma_{1}\gamma_{2}+\frac{1-2\eta \lambda +\eta^{2}\lambda^{2}}{4}\gamma_{1}^{3}\right\}z^{3}+\cdots . \end{array}$$

Similarly, the Eq. (11) gives us

$$\begin{array}{ccc} \frac{D_{q}\left(zD_{q}F_{\zeta ,r\varepsilon ,\varpi }^{n,\rho ,\nu ,q}f\left(z\right)\right)}{D_{q}F_{\zeta ,r\varepsilon ,\varpi }^{n,\rho ,\nu ,q}f\left(z\right)} & = & 1+q\left(1+q\right)a_{2}\tau_{2}z+q\left(1+q\right)\left(\left(1+q+q^{2}\right)a_{3}\tau_{3}-\left(1+q\right)a_{2}^{2}\tau_{2}^{2}\right)z^{2}\\ & & +\;q\left(1+q\right)({\left(1+q\right)}^{2}a_{2}^{3}\tau_{2}^{3}-\left(2+q\right)\left(1+q+q^{2}\right)a_{2}a_{3}\tau_{2}\tau_{3}\\ & & +\;\left(1+q^{2}\right)\left(1+q+q^{2}\right)a_{4}\tau_{4})z^{3}+\cdots . \end{array}$$

By substituting into the Eq. (16) the results are

(19) $$a_{2}=\frac{\lambda \left(1+\eta \right)}{2q\left(1+q\right)\tau_{2}}\gamma_{1},$$

(20) $$a_{3}=\frac{\lambda \left(1+\eta \right)}{2q\left(1+q\right)\left(1+q+q^{2}\right)\tau_{3}}\left\{\gamma_{2}+\left(\frac{\eta \lambda -1}{2}+\frac{\lambda \left(1+\eta \right)}{2q}\right)\gamma_{1}^{2}\right\}$$

and

(21) $$a_{4}=\frac{\lambda \left(1+\eta \right)}{2q\left(1+q\right)\left(1+q^{2}\right)\left(1+q+q^{3}\right)\tau_{4}}\left\{\gamma_{3}+\left(\eta \lambda -1+\frac{\lambda \left(1+\eta \right)}{2}\frac{\left(2+q\right)}{q\left(1+q\right)}\right)\gamma_{1}\gamma_{2}+\Omega_{1}\gamma_{1}^{3}\right\}$$

where

(22) $$\Omega_{1}≔\left\{\begin{array}{c} \frac{1}{4}-\frac{\lambda \eta }{2}+\frac{\eta^{2}\lambda^{2}}{4}+\frac{\lambda \left(1+\eta \right)}{2}\frac{\left(2+q\right)}{q\left(1+q\right)}\\ \times \left(\frac{\eta \lambda -1}{2}+\frac{\lambda \left(1+\eta \right)}{2q}\right)-\frac{\lambda^{2}{\left(1+\eta \right)}^{2}}{4q^{2}} \end{array}\right\}\;.$$

The requested value of inequality $H_{2}\left(1\right)$ is verified by following the process of Theorem 1.

When q→1−andn=0, then $C_{q}\left(1,0,1,\varepsilon ,\varepsilon \right)=C_{q}\left(1,1;1,0,1,\varepsilon ,\varepsilon \right)=C$ and the following outcome occurs:

*Corollary 1. [* 2 *] If* f(z)∈C *, with* f(z) *as in (* 1 *) then*

(23) $$\left|a_{3}-a_{2}^{2}\right|\leq \frac{1}{3}$$

The following findings determine the upper bound of Hankel determinant $H_{2}\left(2\right)$ for the classes $C_{q}\left(\rho ,\nu ,\zeta ,\varpi ,\varepsilon \right)$ and $C_{q}\left(\eta ,\lambda ;\rho ,\nu ,\zeta ,\varpi ,\varepsilon \right)$, respectively:

*Theorem 3. If* $f\left(z\right)\in C_{q}\left(\rho ,\nu ,\zeta ,\varpi ,\varepsilon \right)$ *, with* f(z) *as in (* 1 *) then*

$$H_{2}\left(2\right)=\left|a_{2}a_{4}-a_{3}^{2}\right|\leq \frac{1}{q^{2}{\left(1+q+q^{2}\right)}^{2}\tau_{3}^{2}}.$$

*Proof.* Considering the coefficients $a_{i}\;\left(i=2,3,4\right)$ of Theorem 1 it follows that

$$\begin{array}{ccc} a_{2}a_{4}-a_{3}^{2} & = & \left(\frac{1}{4q^{2}\left(1+q^{2}\right)\left(1+q+q^{2}\right)\tau_{2}\tau_{4}}\right)\gamma_{1}\gamma_{3}\\ & & -\;\left(\frac{\left(q-2-2q^{2}\right)}{8q^{3}\left(1+q^{2}\right)\left(1+q+q^{2}\right)\tau_{2}\tau_{4}}+\frac{\left(1+q^{2}\right)}{4q^{3}{\left(1+q+q^{2}\right)}^{2}\tau_{3}^{2}}\right)\gamma_{1}^{2}\gamma_{2}\\ & & -\;\left(\frac{1}{4q^{2}{\left(1+q+q^{2}\right)}^{2}\tau_{3}^{2}}\right)\gamma_{2}^{2}+\left(\frac{\left(1-q+q^{2}\right)}{16q^{4}\left(1+q+q^{2}\right)\tau_{2}\tau_{4}}-\frac{{\left(1+q^{2}\right)}^{2}}{16q^{4}{\left(1+q+q^{2}\right)}^{2}\tau_{3}^{2}}\right)\gamma_{1}^{4}. \end{array}$$

Utilizing Lemma 2 we attain

$$\begin{array}{ccc} a_{2}a_{4}-a_{3}^{2} & = & \left(\frac{\left(1-q+q^{2}\right)}{16q^{4}\left(1+q+q^{2}\right)\tau_{2}\tau_{4}}-\frac{{\left(1+q^{2}\right)}^{2}}{16q^{4}{\left(1+q+q^{2}\right)}^{2}\tau_{3}^{2}}\right)\gamma_{1}^{4}\\ & & +\;\left(\frac{1}{16q^{2}\left(1+q^{2}\right)\left(1+q+q^{2}\right)\tau_{2}\tau_{4}}\right)\gamma_{1}\{\gamma_{1}^{3}+2\gamma_{1}\left(4-\gamma_{1}^{2}\right)\mu \\ & & -\;\gamma_{1}\left(4-\gamma_{1}^{2}\right)\mu^{2}+2\left(4-\gamma_{1}^{2}\right)\left(1-|\mu {|}^{2}\right)z\}\\ & & -\left(\frac{\left(q-2-2q^{2}\right)}{16q^{3}\left(1+q^{2}\right)\left(1+q+q^{2}\right)\tau_{2}\tau_{4}}+\frac{\left(1+q^{2}\right)}{8q^{3}{\left(1+q+q^{2}\right)}^{2}\tau_{3}^{2}}\right)\gamma_{1}^{2}\left\{\left(\gamma_{1}^{2}+\mu \left(4-\gamma_{1}^{2}\right)\right)\right\}\\ & & -\;\left(\frac{1}{16q^{2}{\left(1+q+q^{2}\right)}^{2}\tau_{3}^{2}}\right)\left\{\gamma_{1}^{4}+2\mu \left(4-\gamma_{1}^{2}\right)\gamma_{1}^{2}+{\left(4-\gamma_{1}^{2}\right)}^{2}\mu^{2}\right\}. \end{array}$$

Setting $\gamma_{1}=\gamma$ and |μ|=ϑ, then

$$\begin{array}{ccc} \left|a_{2}a_{4}-a_{3}^{2}\right| & \leq & \frac{1}{\varphi_{q}^{1}}[\varphi_{q}^{2}\gamma^{4}+2q^{2}\left(1+q+q^{2}\right)\tau_{3}^{2}\gamma \left(4-\gamma^{2}\right)\\ & & +\;\varphi_{q}^{3}\left(4-\gamma^{2}\right)\gamma^{2}\vartheta +(q\left(1+q+q^{2}\right)\tau_{3}^{2}\gamma^{2}+q^{2}\left(1+q^{2}\right)\tau_{2}\tau_{4}\\ & & .\left(4-\gamma^{2}\right)-2q^{2}\left(1+q+q^{2}\right)\tau_{3}^{2}\gamma )\left(4-\gamma^{2}\right)\vartheta^{2}]=J\left(\gamma ,\vartheta \right), \end{array}$$

where $\varphi_{q}^{1}≔16q^{4}\left(1+q^{2}\right){\left(1+q+q^{2}\right)}^{2}\tau_{2}\tau_{3}^{2}\tau_{4},$ $\varphi_{q}^{2}≔\left|\left(1+q^{2}\right){\left(1+q+q^{2}\right)}^{2}\left(\tau_{3}^{2}-\tau_{2}\tau_{4}\right)\right|$

$${\text{and}\;\varphi }_{q}^{3}≔\left|q\left(1+q+q^{2}\right)\left(\left(2+q+2q^{2}\right)\tau_{3}^{2}-2\left(1+q^{2}\right)\tau_{2}\tau_{4}\right)\right|.$$

The partial differentiation of J(γ,ϑ) concerning to ϑ yields the following results:

$$\frac{\partial J\left(\gamma ,\vartheta \right)}{\partial \vartheta }=\frac{1}{\varphi_{q}^{1}}\left[\varphi_{q}^{3}\left(4-\gamma^{2}\right)\gamma^{2}+2\left(q\left(1+q+q^{2}\right)\tau_{3}^{2}\gamma^{2}+q^{2}\left(1+q^{2}\right)\tau_{2}\tau_{4}\left(4-\gamma^{2}\right)-2q^{2}\left(1+q+q^{2}\right)\tau_{3}^{2}\gamma \right)\left(4-\gamma^{2}\right)\vartheta \right]>0.$$

As a result, the function J(γ,ϑ) is an increasing function of ϑ(ϑ∈[0,1]), and we acquire

max{J(γ,ϑ)}=J(γ,1)=G(γ),

where

(24) $$G\left(\gamma \right)≔\left(\frac{1}{\varphi_{q}^{1}}\right)[\begin{array}{c} \left(\varphi_{q}^{2}-\varphi_{q}^{3}-q\left(1+q+q^{2}\right)\tau_{3}^{2}+q^{2}\left(1+q^{2}\right)\tau_{2}\tau_{4}\right)\gamma^{4}\\ +\left(4\varphi_{q}^{3}+4q\left(1+q+q^{2}\right)\tau_{3}^{2}-8q^{2}\left(1+q^{2}\right)\tau_{2}\tau_{4}\right)\gamma^{2}\\ +16q^{2}\left(1+q^{2}\right)\tau_{2}\tau_{4}, \end{array}$$

and

(25) $$\begin{array}{ccc} G^{\prime }\left(\gamma \right) & = & \left(\frac{1}{\varphi_{q}^{1}}\right)[4\left(\varphi_{q}^{2}-\varphi_{q}^{3}-q\left(1+q+q^{2}\right)\tau_{3}^{2}+q^{2}\left(1+q^{2}\right)\tau_{2}\tau_{4}\right)\gamma^{3}\\ & & +\;2\left(4\varphi_{q}^{3}+4q\left(1+q+q^{2}\right)\tau_{3}^{2}-8q^{2}\left(1+q^{2}\right)\tau_{2}\tau_{4}\right)\gamma ]. \end{array}$$

Continue differentiating the function $G_{2}^{\prime }\left(\gamma \right)$ concerning to γ, then

$$\begin{array}{ccc} G^{\prime \prime }\left(\gamma \right) & = & \left(\frac{1}{\varphi_{q}^{1}}\right)[12\left(\varphi_{q}^{2}-\varphi_{q}^{3}-q\left(1+q+q^{2}\right)\tau_{3}^{2}+q^{2}\left(1+q^{2}\right)\tau_{2}\tau_{4}\right)\gamma^{2}\\ & & +\;2\left(4\varphi_{q}^{3}+4q\left(1+q+q^{2}\right)\tau_{3}^{2}-8q^{2}\left(1+q^{2}\right)\tau_{2}\tau_{4}\right)]. \end{array}$$

This indicates that, the highest value of G(γ) happens at γ=0. Therefore, we get

$$\left|a_{2}a_{4}-a_{3}^{2}\right|\leq \frac{1}{q^{2}{\left(1+q+q^{2}\right)}^{2}\tau_{3}^{2}}.$$

*Theorem 4. If* $f\left(z\right)\in C_{q}\left(\eta ,\lambda ;\rho ,\nu ,\zeta ,\varpi ,\varepsilon \right)$ *, with* f(z) *as in (* 1 *) then*

$$H_{2}\left(2\right)=\left|a_{2}a_{4}-a_{3}^{2}\right|\leq \frac{\lambda^{2}{\left(1+\eta \right)}^{2}}{q^{2}{\left(1+q\right)}^{2}{\left(1+q+q^{2}\right)}^{2}\tau_{3}^{2}}.$$

*Proof.* With the assistance of (19), (20) and (21) it will be obtained that

(26) $$a_{2}a_{4}-a_{3}^{2}=\frac{\lambda^{2}{\left(1+\eta \right)}^{2}}{4}\left\{\begin{array}{c} \chi_{1}\gamma_{1}\gamma_{3}+\left(\chi_{1}\left(\eta \lambda -1+\frac{\lambda \left(1+\eta \right)}{2}\frac{\left(2+q\right)}{q\left(1+q\right)}\right)-2\chi_{2}\Omega_{3}\right)\gamma_{1}^{2}\gamma_{2}\\ -\chi_{2}\gamma_{2}^{2}+\left(\chi_{1}\Omega_{1}-\chi_{2}\Omega_{3}^{2}\right)\gamma_{1}^{4} \end{array}\right\}$$

where $\Omega_{1}$ was concluded in (22)

$$\chi_{1}=\frac{1}{q^{2}{\left(1+q\right)}^{2}\left(1+q^{2}\right)\left(1+q+q^{2}\right)\tau_{2}\tau_{4}}\chi_{2}=\frac{1}{q^{2}{\left(1+q\right)}^{2}{\left(1+q+q^{2}\right)}^{2}\tau_{3}^{2}}$$

and

$$\Omega_{3}=\frac{\eta \lambda -1}{2}+\frac{\lambda \left(1+\eta \right)}{2q\tau_{2}}.$$

Employing a similar approach as demonstrated in Theorem 4.3 we achieve the desired result.

When q→1−andn=0, then $C_{q}\left(1,0,1,\varepsilon ,\varepsilon \right)=C_{q}\left(1,1;1,0,1,\varepsilon ,\varepsilon \right)=C$ and the following outcome occurs:

*Corollary 2. [* [30], [43] *] If* f(z)∈C *, with* f(z) *as in (* 1 *) then*

(27) $$\left|a_{2}a_{4}-a_{3}^{2}\right|\leq \frac{1}{8}.$$

*Theorem 3. If*$f\left(z\right)\in C_{q}\left(\rho ,\nu ,\zeta ,\varpi ,\varepsilon \right)$*, with*f(z)*as in (*1*) then*

$$\left|a_{2}a_{3}-a_{4}\right|\leq \frac{\Phi_{q}^{1}}{q^{3}\left(1+q^{2}\right)\left(1+q+q^{2}\right)\tau_{2}\tau_{3}\tau_{4}}$$

where

(28) $$\Phi_{q}^{1}≔\left|\left(1+q^{2}\right)\left(1+q+q^{2}\right)\left(\tau_{4}-\tau_{2}\tau_{3}\right)\right|.$$

*Proof.* Simplifying from (12) to (14) of Theorem 1 we procure

(29) $$\begin{array}{ccc} a_{2}a_{3}-a_{4} & = & \left(\frac{\left(1+q^{2}\right)}{8q^{3}\left(1+q+q^{2}\right)\tau_{2}\tau_{3}}-\frac{\left(1-q+q^{2}\right)}{8q^{3}\left(1+q+q^{2}\right)\tau_{4}}\right)\gamma_{1}^{3}-\frac{1}{2q\left(1+q^{2}\right)\left(1+q+q^{2}\right)\tau_{4}}\gamma_{3}\\ & & +\;\left(\frac{1}{4q^{2}\left(1+q+q^{2}\right)\tau_{2}\tau_{3}}-\frac{\left(2q^{2}-q+2\right)}{4q^{2}\left(1+q^{2}\right)\left(1+q+q^{2}\right)\tau_{4}}\right)\gamma_{1}\gamma_{2}. \end{array}$$

Employing Lemma 2 we attain

$$\begin{array}{ccc} a_{2}a_{3}-a_{4} & = & \left(\frac{\left(1+q^{2}\right)}{8q^{3}\left(1+q+q^{2}\right)\tau_{2}\tau_{3}}-\frac{\left(1-q+q^{2}\right)}{8q^{3}\left(1+q+q^{2}\right)\tau_{4}}\right)\gamma_{1}^{3}\\ & & -\;\frac{1}{8q\left(1+q^{2}\right)\left(1+q+q^{2}\right)\tau_{4}}\left[\gamma_{1}^{3}+2\gamma_{1}\left(4-\gamma_{1}^{2}\right)\mu -\gamma_{1}\left(4-\gamma_{1}^{2}\right)\mu^{2}+2\left(4-\gamma_{1}^{2}\right)\left(1-|\mu {|}^{2}\right)z\right]\\ & & +\;\left(\frac{1}{8q^{2}\left(1+q+q^{2}\right)\tau_{2}\tau_{3}}-\frac{\left(2q^{2}-q+2\right)}{8q^{2}\left(1+q^{2}\right)\left(1+q+q^{2}\right)\tau_{4}}\right)\gamma_{1}\left\{\gamma_{1}^{2}+\mu \left(4-\gamma_{1}^{2}\right)\right\}. \end{array}$$

Afterwards, assuming $\gamma_{1}=\gamma$ and using |μ|=ϑ, we obtain

$$\left|a_{2}a_{3}-a_{4}\right|\leq K\left(\gamma ,\vartheta \right),$$

where

$$\begin{array}{ccc} K\left(\gamma ,\vartheta \right) & & ≔\left(\frac{1}{8q^{3}\left(1+q^{2}\right)\left(1+q+q^{2}\right)\tau_{2}\tau_{3}\tau_{4}}\right)[\Phi_{q}^{1}\gamma^{3}+\Phi_{q}^{2}\gamma \left(4-\gamma^{2}\right)\vartheta \\ & & +2q^{2}\tau_{2}\tau_{3}\left(4-\gamma^{2}\right)+q^{2}\tau_{2}\tau_{3}\left(\gamma -2\right)\left(4-\gamma^{2}\right)\vartheta^{2}], \end{array}$$

with $\Phi_{q}^{2}≔\left|q\left(1+q^{2}\right)\tau_{4}-q\left(2+q+2q^{2}\right)\tau_{2}\tau_{3}\right|$ and $\Phi_{q}^{1}$ well-known in (28).

Upon differentiating the function K(γ,ϑ) concerning to ϑ, we obtain

$$K^{\prime }\left(\gamma ,\vartheta \right)=\left(\frac{1}{8q^{3}\left(1+q^{2}\right)\left(1+q+q^{2}\right)\tau_{2}\tau_{3}\tau_{4}}\right)[\Phi_{q}^{2}\gamma \left(4-\gamma^{2}\right)+2q^{2}\tau_{2}\tau_{3}\left(\gamma -2\right)\left(4-\gamma^{2}\right)\vartheta ]>0.$$

As a result, the function K(γ,ϑ) is an increasing function of ϑ(ϑ∈[0,1]), and we acquire

K(γ,ϑ)≤K(γ,1).

Subsequently,

max{K(γ,ϑ)}=K(γ,1)≤L(γ),

where

$$L\left(\gamma \right)≔\left(\frac{1}{8q^{3}\left(1+q^{2}\right)\left(1+q+q^{2}\right)\tau_{2}\tau_{3}\tau_{4}}\right)\left[\left(\Phi_{q}^{1}-\Phi_{q}^{2}-q^{2}\tau_{2}\tau_{3}\right)\gamma^{3}+\left(4\Phi_{q}^{2}+4q^{2}\tau_{2}\tau_{3}\right)\gamma \right].$$

Since 0≤γ≤2, which indicates that γ=2 is the maximal point, so

$$L\left(\gamma \right)\leq \frac{\Phi_{q}^{1}}{q^{3}\left(1+q^{2}\right)\left(1+q+q^{2}\right)\tau_{2}\tau_{3}\tau_{4}}$$

this relates to the intended limit, γ=2, and ϑ=1.

We can deduce the bound values of the inequality $|a_{2}a_{3}-a_{4}|$ for the class $C_{q}\left(\eta ,\lambda ;\rho ,\nu ,\zeta ,\varpi ,\varepsilon \right)$ in view of the previous theorem

*Theorem 6. If*$f\left(z\right)\in C_{q}\left(\eta ,\lambda ;\rho ,\nu ,\zeta ,\varpi ,\varepsilon \right)$*, with*f(z)*as in (*1*) then*

(30) $$\left|a_{2}a_{3}-a_{4}\right|\leq \frac{\left(1+\eta \right)\lambda \Gamma_{q}^{1}}{q^{3}\left(1+q^{2}\right){\left(1+q\right)}^{2}\left(1+q+q^{2}\right)\tau_{2}\tau_{3}\tau_{4}}$$

where

$$\begin{array}{ccc} \Gamma_{q}^{1}≔ & & |\left(1+q^{2}\right)\left(1+\eta \right)\left(q\eta \lambda^{2}+\left(1+\eta \right)\lambda^{2}\right)\tau_{4}-(q\left(2+q\right)\left(1+\eta \right)\eta \lambda^{2}\\ & & +\;\left(1+\eta \left(2+\left(1+q^{2}+q^{3}\right)\eta \right)\right)\lambda^{2})\tau_{2}\tau_{3}|. \end{array}$$

*Proof.* Simplifying from (19) to (21) of Theorem 3 we procure

(31) $$\begin{array}{ccc} a_{2}a_{3}-a_{4} & = & \left(\frac{{\left(1+\eta \right)}^{2}\lambda \left(q\left(\eta \lambda^{2}-\lambda \right)+\left(1+\eta \right)\lambda^{2}\right)}{8q^{3}{\left(1+q\right)}^{2}\left(1+q+q^{2}\right)\tau_{2}\tau_{3}}-\frac{\left(1+\eta \right)\lambda Y_{q}^{1}}{8q^{3}\left(1+q^{2}\right){\left(1+q\right)}^{2}\left(1+q+q^{2}\right)\tau_{4}}\right)\gamma_{1}^{3}\\ & & -\;\left(\frac{\left(1+\eta \right)\lambda }{2q\left(1+q^{2}\right)\left(1+q\right)\left(1+q+q^{2}\right)\tau_{4}}\right)\gamma_{3}+(\frac{{\left(1+\eta \right)}^{2}\lambda^{2}}{4q^{2}{\left(1+q\right)}^{2}\left(1+q+q^{2}\right)\tau_{2}\tau_{3}}\\ & & -\;\frac{\left(1+\eta \right)\lambda \left(2q\left(1+q\right)\left(\eta \lambda -1\right)+\left(2+q\right)\left(1+\eta \right)\lambda \right)}{4q^{2}\left(1+q^{2}\right){\left(1+q\right)}^{2}\left(1+q+q^{2}\right)\tau_{4}})\gamma_{1}\gamma_{2}, \end{array}$$

where

$$Y_{q}^{1}≔2q\left(1+\eta \right)\left(\eta \lambda^{2}-\lambda \right)+{\left(1+\eta \right)}^{2}\lambda^{2}+q^{3}\left(1-2\eta \lambda +\eta^{2}\lambda^{2}\right)$$

$$+q^{2}\left(1-2\eta \lambda +\left(1+\eta \right)\eta \lambda^{2}+\lambda \left(-1+\eta \left(-1+\eta \lambda \right)\right)\right).$$

By continuing with the analogous technique of Theorem 5 the inequality (30) is derived.

When q→1−andn=0, then $C_{q}\left(1,0,1,\varepsilon ,\varepsilon \right)=C_{q}\left(1,1;1,0,1,\varepsilon ,\varepsilon \right)=C$ and the following outcome occurs:

*Corollary 3. [6] If* f(z)∈C *, with* f(z) *as in (1) then*

(32) $$\left|a_{2}a_{3}-a_{4}\right|\leq \frac{1}{6}.$$

## Toeplitz determinants findings

This part considers the bound values of the second $T_{2}\left(2\right)$ and third $T_{3}\left(1\right)$ orders inequalities of Toeplitz matrix.

*Theorem 7. If* $f\left(z\right)\in C_{q}\left(\rho ,\nu ,\zeta ,\varpi ,\varepsilon \right)$ *, with* f(z) *as in (* 1 *) then*

(33) $$T_{2}\left(2\right)=\left|a_{3}^{2}-a_{2}^{2}\right|\leq \frac{1}{q^{2}{\left(1+q+q^{2}\right)}^{2}\tau_{3}^{2}}\left(\frac{{\left(1+q\right)}^{2}\left(1+q^{2}\right)}{q^{2}}+\frac{{\left(1+q+q^{2}\right)}^{2}\tau_{3}^{2}}{\tau_{2}^{2}}\right).$$

*Proof.* In Theorem 1 given the values $a_{2}$ and $a_{3}$, we have

$$\left|a_{3}^{2}-a_{2}^{2}\right|=\left|{\left(\frac{1}{2q\left(1+q+q^{2}\right)\tau_{3}}\gamma_{2}+\frac{\left(1+q^{2}\right)}{4q^{2}\left(1+q+q^{2}\right)\tau_{3}}\gamma_{1}^{2}\right)}^{2}-\frac{1}{4q^{2}\tau_{2}^{2}}\gamma_{1}^{2}\right|$$

and

$$\left|a_{3}^{2}-a_{2}^{2}\right|=\frac{1}{4q^{2}{\left(1+q+q^{2}\right)}^{2}\tau_{3}^{2}}\left|\gamma_{2}^{2}+\frac{{\left(1+q^{2}\right)}^{2}}{4q^{2}}\gamma_{1}^{4}+\left(\frac{\left(1+q^{2}\right)\tau_{2}^{2}\gamma_{2}-q{\left(1+q+q^{2}\right)}^{2}\tau_{3}^{2}}{q\tau_{2}^{2}}\right)\gamma_{1}^{2}\right|.$$

Employing Lemma 2 with $\gamma_{1}\leq 2$, we attain

$$\left|a_{3}^{2}-a_{2}^{2}\right|=\frac{1}{4q^{2}{\left(1+q+q^{2}\right)}^{2}\tau_{3}^{2}}\left|\frac{1}{4}\mu^{2}X^{2}+\frac{\left(1+q^{2}\right)}{2q}\gamma_{1}^{2}\mu X\;+\frac{{\left(1+q\right)}^{2}\left(1+q^{2}\right)}{4q^{2}}\gamma_{1}^{4}-\frac{{\left(1+q+q^{2}\right)}^{2}\tau_{3}^{2}}{\tau_{2}^{2}}\gamma_{1}^{2}\right|,$$

where $X≔(4-\gamma_{1}^{2}).$

Afterwards, assuming $\gamma_{1}=\gamma$ with 0≤γ≤2, and using |μ|=ϑ, we obtain

(34) $$\begin{array}{c} \left|a_{3}^{2}-a_{2}^{2}\right|\leq G_{q}\left(\vartheta \right)≔\frac{1}{4q^{2}{\left(1+q+q^{2}\right)}^{2}\tau_{3}^{2}}\left(\left|\frac{{\left(1+q\right)}^{2}\left(1+q^{2}\right)}{4q^{2}}\gamma^{4}-\frac{{\left(1+q+q^{2}\right)}^{2}\tau_{3}^{2}}{\tau_{2}^{2}}\gamma^{2}\right|+\frac{1}{4}\vartheta^{2}X^{2}+\frac{\left(1+q^{2}\right)}{2q}\gamma^{2}\vartheta X\right), \end{array}$$

with $X=4-\gamma^{2}.$

Upon differentiating the function $G_{q}\left(\vartheta \right)$ concerning to ϑ(0≤ϑ≤1), we consider that

$$\frac{\partial G_{q}}{\partial \vartheta }=\frac{1}{4q^{2}{\left(1+q+q^{2}\right)}^{2}\tau_{3}^{2}}\left(\frac{1}{2}\vartheta X^{2}+\frac{\left(1+q^{2}\right)}{2q}\gamma^{2}X\right)>0.$$

Consequently, we procure that the function G(ϑ) is an increasing function of ϑ with ϑ=1.

$$max\left\{G_{q}\left(\vartheta \right)\right\}=G_{q}\left(1\right),$$

where

$$\left|a_{3}^{2}-a_{2}^{2}\right|\leq \frac{1}{4q^{2}{\left(1+q+q^{2}\right)}^{2}\tau_{3}^{2}}\left(\left|\frac{{\left(1+q\right)}^{2}\left(1+q^{2}\right)}{4q^{2}}\gamma^{4}-\frac{{\left(1+q+q^{2}\right)}^{2}\tau_{3}^{2}}{\tau_{2}^{2}}\gamma^{2}\right|+\frac{1}{4}X^{2}+\frac{\left(1+q^{2}\right)}{2q}\gamma^{2}X\right).$$

γ=2 is the maximal point since 0≤γ≤2. The desired outcome then materializes.

We derive now the inequality $|a_{3}^{2}-a_{2}^{2}|$ for the class $C_{q}\left(\eta ,\lambda ;\rho ,\nu ,\zeta ,\varpi ,\varepsilon \right)$ considering the preceding theorem.

*Theorem 8. If*$f\left(z\right)\in C_{q}\left(\eta ,\lambda ;\rho ,\nu ,\zeta ,\varpi ,\varepsilon \right)$*, with*f(z)*as in (1.1) then*

(35) $$T_{2}\left(2\right)=\left|a_{3}^{2}-a_{2}^{2}\right|\leq \frac{{\left(1+\eta \right)}^{2}\lambda^{2}}{4q^{2}{\left(1+q+q^{2}\right)}^{2}{\left(1+q+q^{2}\right)}^{2}\tau_{3}^{2}}\left(\frac{\Psi_{\eta }^{\lambda }}{4q^{2}}+\frac{{\left(1+q+q^{2}\right)}^{2}\tau_{3}^{2}}{\tau_{2}^{2}}\right),$$

where $\Psi_{\eta }^{\lambda }≔\left|q\left(1+\eta \right)\eta \lambda^{2}+q^{2}\left(\eta \lambda^{2}-1\right)+{\left(1+\eta \right)}^{2}\lambda^{2}\right|.$

*Proof.* In Theorem 3 given the values $a_{2}$ and $a_{3}$, we have

$$\left|a_{3}^{2}-a_{2}^{2}\right|=\left|{\left(\frac{\lambda \left(1+\eta \right)}{2q\left(1+q\right)\left(1+q+q^{2}\right)\tau_{3}}\left\{\gamma_{2}+\left(\frac{\eta \lambda -1}{2}+\frac{\lambda \left(1+\eta \right)}{2q}\right)\gamma_{1}^{2}\right\}\right)}^{2}-\frac{\lambda^{2}{\left(1+\eta \right)}^{2}}{4q^{2}{\left(1+q\right)}^{2}\tau_{2}^{2}}\gamma_{1}^{2}\right|$$

and

$$\left|a_{3}^{2}-a_{2}^{2}\right|=\frac{{\left(1+\eta \right)}^{2}\lambda^{2}}{4q^{2}{\left(1+q\right)}^{2}{\left(1+q+q^{2}\right)}^{2}\tau_{3}^{2}}\left|\gamma_{2}^{2}+\frac{\psi_{1,\eta }^{\lambda }}{4q^{2}}\gamma_{1}^{4}+\left(\frac{\psi_{2,\eta }^{\lambda }\tau_{2}^{2}\gamma_{2}-q{\left(1+q+q^{2}\right)}^{2}\tau_{3}^{2}}{q\tau_{2}^{2}}\right)\gamma_{1}^{2}\right|,$$

where $\psi_{1,\eta }^{\lambda }≔2q\left(1+\eta \right)\left(\eta \lambda^{2}-\lambda \right)+{\left(1+\eta \right)}^{2}\lambda^{2}+q^{2}\left(1+\eta \lambda^{2}-2\eta \lambda \right)$ and $\psi_{2,\eta }^{\lambda }≔q\eta \lambda -q+\lambda +\eta \lambda .$

Employing Lemma 2 with $\gamma_{1}\leq 2$, we attain

$$\left|a_{3}^{2}-a_{2}^{2}\right|=\frac{{\left(1+\eta \right)}^{2}\lambda^{2}}{4q^{2}{\left(1+q+q^{2}\right)}^{2}{\left(1+q+q^{2}\right)}^{2}\tau_{3}^{2}}\left|\frac{1}{4}\mu^{2}X^{2}+\frac{\psi_{2,\eta }^{\lambda }}{2q}\gamma_{1}^{2}\mu X+\frac{\Psi_{\eta }^{\lambda }}{4q^{2}}\gamma_{1}^{4}-\frac{{\left(1+q+q^{2}\right)}^{2}\tau_{3}^{2}}{\tau_{2}^{2}}\gamma_{1}^{2}\right|,$$

where $X≔\left(4-\gamma_{1}^{2}\right){\;\text{and}\;\Psi }_{\eta }^{\lambda }≔q\left(1+\eta \right)\eta \lambda^{2}+q^{2}\left(\eta \lambda^{2}-1\right)+{\left(1+\eta \right)}^{2}\lambda^{2}.$

The inequality (34) is derived by carrying out the same procedure of Theorem 7.

*Theorem 9. If* $f\left(z\right)\in C_{q}\left(\rho ,\nu ,\zeta ,\varpi ,\varepsilon \right)$ *, with* f(z) *as in (1.1) then*

$$T_{3}\left(1\right)=\left|\begin{array}{ccc} 1 & a_{2} & a_{3}\\ a_{2} & 1 & a_{2}\\ a_{3} & a_{2} & 1 \end{array}\right|\leq \frac{q^{4}\tau_{2}^{2}\tau_{3}^{2}+2q^{2}\tau_{3}^{2}+\tau_{2}^{2}+2\tau_{3}}{q^{4}\tau_{2}^{2}\tau_{3}^{2}}.$$

*Proof.* In Theorem 1 given the values $a_{2}$ and $a_{3}$, we have

$$\begin{array}{cc} T_{3}\left(1\right) & =\left|1+2a_{2}^{2}\left(a_{3}-1\right)-a_{3}^{2}\right|\\ & =\left|1+\Lambda_{q}^{1}\gamma_{1}^{2}\gamma_{2}+\Lambda_{q}^{2}\gamma_{1}^{4}-\frac{\gamma_{1}^{2}}{2q^{2}\tau_{2}^{2}}-\frac{\gamma_{2}^{2}}{4q^{2}{\left(1+q+q^{2}\right)}^{2}\tau_{3}^{2}}\right|, \end{array}$$

where

$$\Lambda_{q}^{1}≔\frac{\left(1+q+q^{2}\right)\tau_{3}-\left(1+q^{2}\right)\tau_{2}^{2}}{4q^{3}{\left(1+q+q^{2}\right)}^{2}\tau_{2}^{2}\tau_{3}^{2}}$$

and

$$\Lambda_{q}^{2}≔\frac{2\left(1+q^{2}\right)\left(1+q+q^{2}\right)\tau_{3}-{\left(1+q^{2}\right)}^{2}\tau_{2}^{2}}{16q^{4}{\left(1+q+q^{2}\right)}^{2}\tau_{2}^{2}\tau_{3}^{2}}.$$

Considering Lemma 2 we observe that

$$T_{3}\left(1\right)=\left|1+\frac{2\tau_{3}-\tau_{2}^{2}}{16q^{4}\tau_{2}^{2}\tau_{3}^{2}}\gamma_{1}^{4}-\frac{\gamma_{1}^{2}}{2q^{2}\tau_{2}^{2}}-\frac{1}{16q^{2}{\left(1+q+q^{2}\right)}^{2}\tau_{3}^{2}}\mu^{2}X^{2}+\Lambda_{q}^{3}\gamma_{1}^{2}\mu X\right|,$$

where $X=(4-\gamma_{1}^{2})$ with

$$\Lambda_{q}^{3}=\frac{\tau_{3}-\tau_{2}^{2}}{8q^{3}\left(1+q+q^{2}\right)\tau_{2}^{2}\tau_{3}^{2}}.$$

Afterwards, assuming $\gamma_{1}=\gamma$ with 0≤γ≤2, and using |μ|=ϑ, we deduce that

$$T_{3}\left(1\right)\leq W_{q}\left(\vartheta \right)=\left|1+\frac{2\tau_{3}-\tau_{2}^{2}}{16q^{4}\tau_{2}^{2}\tau_{3}^{2}}\gamma^{4}-\frac{\gamma^{2}}{2q^{2}\tau_{2}^{2}}\right|+\frac{1}{16q^{2}{\left(1+q+q^{2}\right)}^{2}\tau_{3}^{2}}\vartheta^{2}X^{2}+\Lambda_{q}^{3}\gamma_{1}^{2}\vartheta X.$$

Upon differentiating the function $W_{q}\left(\vartheta \right)$ concerning to ϑ(0≤ϑ≤1), we consider that

$$\frac{\partial W_{q}\left(\vartheta \right)}{\partial \delta }>0.$$

This proves that the function $W_{q}\left(\vartheta \right)$ increses and reaches its greatest value at ϑ=1.

$$max\left\{W_{q}\left(\vartheta \right)\right\}=W_{q}\left(1\right),$$

where

$$T_{3}\left(1\right)\leq \left|1+\frac{2\tau_{3}-\tau_{2}^{2}}{16q^{4}\tau_{2}^{2}\tau_{3}^{2}}\gamma^{4}-\frac{\gamma^{2}}{2q^{2}\tau_{2}^{2}}\right|+\frac{1}{16q^{2}{\left(1+q+q^{2}\right)}^{2}\tau_{3}^{2}}X^{2}+\Lambda_{q}^{3}\gamma_{1}^{2}X.$$

The maximal point is γ=2 since 0≤γ≤2. The desired result occurs.

*Theorem 10. If*$f\left(z\right)\in C_{q}\left(\eta ,\lambda ;\rho ,\nu ,\zeta ,\varpi ,\varepsilon \right)$*, with*f(z)*as in (*1*) then*

(36) $$T_{3}\left(1\right)=\left|1+2a_{2}^{2}\left(a_{3}-1\right)-a_{3}^{2}\right|\leq \left(1+\Sigma_{q;\eta }^{\lambda }+\frac{2{\left(1-\eta \right)}^{2}\lambda^{2}}{q^{2}{\left(1+q\right)}^{2}\tau_{2}^{2}}\right),$$

where

$$\begin{array}{ccc} \Sigma_{q;\eta }^{\lambda } & ≔ & \frac{1}{16q^{4}{\left(1+q\right)}^{3}{\left(1+q+q^{2}\right)}^{2}\psi_{2}^{2}\psi_{3}^{2}}|2\left(1+q+q^{2}\right){\left(1+\eta \right)}^{3}\lambda^{2}\left(q\eta \lambda^{2}+\left(1+\eta \right)\lambda^{2}\right)\psi_{3}\\ & & -\;\left(1+q\right)\left({\left(\eta^{2}-1\right)}^{2}\lambda^{4}+2q\left(1+\eta \right)\lambda^{2}\left({\left(\eta -1\right)}^{2}\eta \lambda^{2}+4\eta \lambda \right)+q^{2}\left({\left(\eta -1\right)}^{2}\eta \lambda^{4}+8\eta^{2}\lambda^{3}-4\eta \lambda^{2}\right)\right)\psi_{2}^{2}|. \end{array}$$

*Proof.* In Theorem 3 with $a_{2}$ and $a_{3}$, we have

$$\begin{array}{c} T_{3}\left(1\right)=\left|1+\Lambda_{q}^{4}\gamma_{1}^{2}\gamma_{2}+\Lambda_{q}^{5}\gamma_{1}^{4}-\frac{{\left(1-\eta \right)}^{2}\lambda^{2}}{2q^{2}{\left(1+q\right)}^{2}\tau_{2}^{2}}\gamma_{1}^{2}-\frac{{\left(1+\eta \right)}^{2}\lambda^{2}}{4q^{2}{\left(1+q\right)}^{2}{\left(1+q+q^{2}\right)}^{2}\tau_{3}^{2}}\gamma_{2}^{2}\right|, \end{array}$$

where

$$\Lambda_{q}^{4}≔\frac{\lambda {\left(1+\eta \right)}^{2}\left(\left(1+q+q^{2}\right)\left(1+\eta \right)\lambda^{2}\tau_{3}-\left(1+q\right)\left(q\left(\eta \lambda^{2}-\lambda \right)+\left(1+\eta \right)\lambda^{2}\right)\tau_{2}^{2}\right)}{4q^{3}{\left(1+q\right)}^{3}{\left(1+q+q^{2}\right)}^{2}\tau_{2}^{2}\tau_{3}^{2}}$$

and

$$\begin{array}{ccc} \Lambda_{q}^{5} & ≔ & \frac{1}{16q^{4}{\left(1+q\right)}^{3}{\left(1+q+q^{2}\right)}^{2}\tau_{2}^{2}\tau_{3}^{2}}(2\left(1+q+q^{2}\right){\left(1+\eta \right)}^{3}\lambda^{2}\left(q\left(\eta \lambda^{2}-\lambda \right)+\left(1+\eta \right)\lambda^{2}\right)\tau_{3}-\left(1+q\right)\\ & & {\left(1-\eta \right)}^{2}\left(q\left(\eta \lambda^{2}-\lambda \right)+{\left(+\left(1+\eta \right)\lambda^{2}\right)}^{2}\tau_{2}^{2}\right). \end{array}$$

The inequality (35) is produced following the same technique as Theorem 9.

## Application by pascal distribution

The Taylor series whose their coeffiecients representing Pascal distribution probabilities was provided by Murugusundaramoorthy et al. [44], which is formulated as

$$P_{\rho }^{\varepsilon }f\left(z\right):z+\sum_{i=2}^{\infty }\left(\begin{array}{c} i+\varepsilon -2\\ \varepsilon -1 \end{array}\right)\rho^{i-1}{\left(1-\rho \right)}^{\varepsilon }a_{i}z^{i}=z+\varepsilon \rho {\left(1-\rho \right)}^{\varepsilon }a_{2}z^{2}+\frac{\varepsilon \left(\varepsilon +1\right)}{2}\rho^{2}{\left(1-\rho \right)}^{\varepsilon }a_{3}z^{3}+\cdots .$$

Replacing the operator $F_{\zeta ,r\varepsilon ,\varpi }^{n,\rho ,\nu ,q}f\left(z\right)$ by the operator $P_{\rho }^{\varepsilon }f\left(z\right),$ then we get the following coefficients:

*Theorem 9. If*$P_{\rho }^{\varepsilon }f\left(z\right)\in C_{q}\left(\rho ,\nu ,\zeta ,\varpi ,\varepsilon \right)$*, then*

$$\left|a_{2}\right|\leq \frac{1}{q\varepsilon \rho {\left(1-\rho \right)}^{\varepsilon }},$$

$$\left|a_{3}\right|\leq \frac{2}{q^{2}\varepsilon \left(\varepsilon +1\right)\rho^{2}{\left(1-\rho \right)}^{\varepsilon }}$$

and

$$\left|a_{4}\right|\leq \frac{3\left(1+q\right)\left(q^{3}+q+1\right)}{q^{3}\left(1+q^{2}\right)\left(1+q+q^{2}\right)\varepsilon \left(\varepsilon +1\right)\left(\varepsilon +2\right)\rho^{3}{\left(1-\rho \right)}^{\varepsilon }}.$$

*Theorem 10. If*$P_{\rho }^{\varepsilon }f\left(z\right)\in C_{q}\left(\eta ,\lambda ;\rho ,\nu ,\zeta ,\varpi ,\varepsilon \right)$, *then*

$$|a_{2}|\leq \frac{\lambda \left(1+\eta \right)}{q\left(1+q\right)\varepsilon \rho {\left(1-\rho \right)}^{\varepsilon }},$$

$$\left|a_{3}\right|\leq \frac{2\lambda \left(1+\eta \right)\left(q\eta \lambda +\lambda \left[1+\eta \right]\right)}{q^{2}\left(1+q\right)\left(1+q+q^{2}\right)\varepsilon \left(\varepsilon +1\right)\rho^{2}{\left(1-\rho \right)}^{\varepsilon }}$$

and

$$\left|a_{4}\right|\leq \frac{3\left(1+\eta \right)\lambda \left(q\left(2+q\right)\lambda \left(1+\eta \right)\left(q\eta \lambda +\lambda \left(1+\eta \right]\right)+q\left(1+q\right)\left(q^{2}\left(2\eta \lambda +\eta \lambda \left(-2+\eta \lambda \right)\right)-\lambda^{2}{\left(1+\eta \right)}^{2}\right)\right)}{q^{4}{\left(1+q\right)}^{2}\left(1+q^{2}\right)\left(1+q+q^{3}\right)\varepsilon \left(\varepsilon +1\right)\left(\varepsilon +2\right)\rho^{3}{\left(1-\rho \right)}^{\varepsilon }}.$$

## Conclusion

This work's primary goal was to improve the previously established limits of the Hankel and Toeplitz determinants for the classes of analytic convex functions (see [[30], [45], [46]]). To present new findings, we used q-calculus. This study may encourage the adoption of more operators. Furthermore, the supplied boundary values of the inequalities might serve as a foundation for examining the requirements for the operator's univalence presented in this work. Using the operator $F_{\zeta ,r\varepsilon ,\varpi }^{n,\rho ,\nu ,q}f\left(z\right)$ given in Eq. (5), further study may include the creation of new classes of analytic functions. Moreover, other ideas on neighbourhoods, differential subordination, and the Fekete-Szegö problem can be studied.

## Limitations

Not applicable.

## Ethics authors statements

The platforms’ data redistribution policies were complied with.

## Funding statement

This research received no external funding.

## CRediT authorship contribution statement

**Sarem H. Hadi:** Conceptualization, Methodology, Writing – original draft, Visualization, Investigation, Software. **Timilehin Gideon Shaba:** Conceptualization, Methodology, Writing – original draft. **Zainab S. Madhi:** Visualization, Investigation, Software, Writing – original draft. **Maslina Darus:** Conceptualization, Methodology, Writing – original draft. **Alina Alb Lupaş:** Visualization, Investigation, Software, Writing – original draft. **Fairouz Tchier:** Visualization, Investigation, Software, Writing – original draft.

## Declaration of competing interest

The authors declare that they have no known competing financial interests or personal relationships that could have appeared to influence the work reported in this paper.

## Data Availability

No data was used for the research described in the article.
